# Single-Walled Carbon Nanotubes as Fluorescent Probes for Monitoring the Self-Assembly and Morphology of Peptide/Polymer Hybrid Hydrogels

**DOI:** 10.1021/acs.nanolett.2c01587

**Published:** 2022-10-19

**Authors:** Verena Wulf, Gili Bisker

**Affiliations:** † Department of Biomedical Engineering, Faculty of Engineering, Tel-Aviv University, Tel Aviv 6997801, Israel; ‡ The Center for Physics and Chemistry of Living Systems, Tel-Aviv University, Tel Aviv 6997801, Israel; § Center for Nanoscience and Nanotechnology, Tel-Aviv University, Tel Aviv 6997801, Israel; ∥ Center for Light Matter Interaction, Tel-Aviv University, Tel Aviv 6997801, Israel

**Keywords:** low-molecular-weight gelator, Fmoc-diphenylalanine, single-walled carbon nanotubes, self-assembly, fluorescence imaging, near-infrared fluorescent sensors

## Abstract

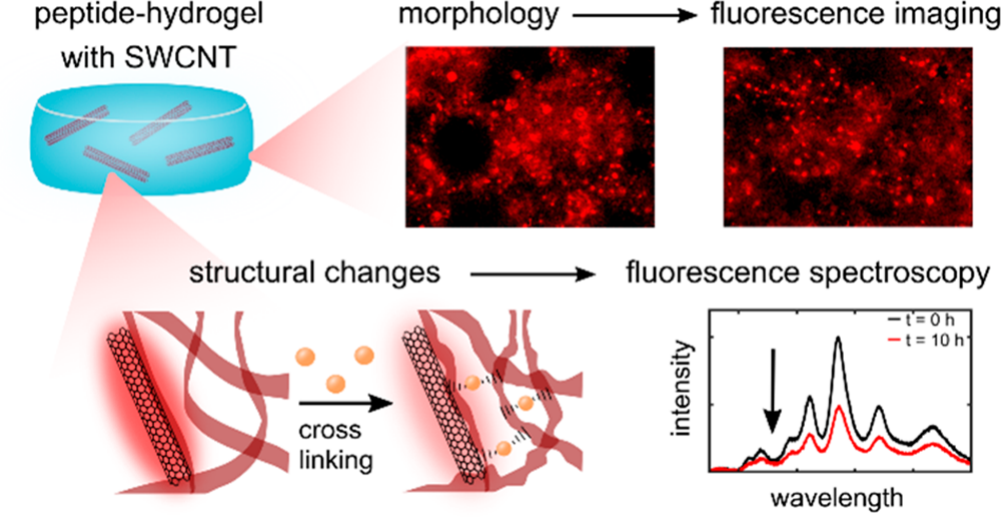

Hydrogels formed via supramolecular self-assembly of fluorenylmethyloxycarbonyl (Fmoc)-conjugated amino acids provide excellent scaffolds for 3D cell culture, tissue engineering, and tissue recovery matrices. Such hydrogels are usually characterized by rheology or electron microscopy, which are invasive and cannot provide real-time information. Here, we incorporate near-infrared fluorescent single-walled carbon nanotubes (SWCNTs) into Fmoc-diphenylalanine hydrogels as fluorescent probes, reporting in real-time on the morphology and time-dependent structural changes of the self-assembled hydrogels in the transparency window of biological tissue. We further demonstrate that the gelation process and structural changes upon the addition of cross-linking ions are transduced into spectral modulations of the SWCNT-fluorescence. Moreover, morphological differences of the hydrogels induced by polymer additives are manifested in unique features in fluorescence images of the incorporated SWCNTs. SWCNTs can thus serve as optical probes for noninvasive, long-term monitoring of the self-assembly gelation process and the fate of the resulting peptide hydrogel during long-term usage.

Peptide hydrogels find applications not only as matrices for *in vitro* 3D cell cultures^1−3^ but also as *in vivo* biomimetic scaffolds like injectable hydrogels for tissue engineering,^4,5^ tissue repair, and recovery.^6−9^ In contrast to polymeric hydrogels, peptide hydrogel matrices are formed by physical interactions between specific amino acids or oligopeptides.^10,11^ These amino acids or peptides have limited solubility in water and prefer to self-assemble into long fibrillary nanostructures that can entangle to form a self-supported hydrogel.^12,13^ Due to their fibrillary structures that mimic the morphology of fibrous proteins of the extracellular matrix, e.g., collagen, peptide hydrogels offer excellent matrices for tissue engineering, repair, or wound healing.^14,15^ Chondrocytes embedded into peptide hydrogels were shown to produce collagen, replacing the peptide hydrogel as a temporary matrix and leading to tissue repair.^6,7^ For each application, different morphological structures and mechanical strengths of the hydrogels are required, mainly depending on the chemical composition of the gelator. The library of amino acids reported to self-assemble includes natural α-amino acids, conjugated amino acids, e.g., naphthalene conjugates or fluorenylmethyloxycarbonyl (Fmoc) conjugates^16−19^ and also unnatural amino acids.^20^ To further tune the properties of the peptide hydrogels, additives like inorganic nanoparticles^21^ or polymers^9,15,22−25^ are added, where polymers especially find broad relevance in biomedical applications.^26^

One of the most studied conjugated amino acids forming self-supported hydrogels is Fmoc-diphenylalanine (FmocFF).^9,19,27−29^ FmocFF is soluble in organic solvents and in water under basic conditions. Hydrogelation can be induced by lowering the pH of a basic aqueous solution (pH-switch) of FmocFF^30,31^ or changing the polarity of an organic solvent by adding water (solvent-switch).^27,32^ Further, Ca^2+^-ions are reported to induce the self-assembly of FmocFF as cross-linkers.^33,34^ The stability of the resulting gel and its morphology is highly dependent on the chosen conditions of the manufacturing process.^31,35^

The characterization of the gelation process and the changes in mechanical properties of the peptide hydrogels during their usage is mostly done via rheometry, which does not allow measuring the gels in their designated environment, e.g., integrated into tissue. Morphological properties of the hydrogels are measured via electron microscopy or atomic force microscopy. These methods are highly invasive and do not measure the gels as a whole. In contrast, fluorescence imaging and/or spectroscopy are noninvasive methods to characterize real-time processes. Traditional organic fluorescent dyes integrated into hydrogels often suffer from photobleaching, posing a challenge on monitoring hydrogels whose aging time is reported to be up to 3 days and that are used afterward for several days or weeks.^22,36−39^ Thus, there is still a need for noninvasive, long-term means to monitor the self-assembly processes of peptide hydrogels and structural changes inside the gels upon condition modifications, preferably in deep tissue.

Single-walled carbon nanotubes (SWCNTs) can be seen as graphene sheets rolled up into nanotubes, resulting in nanostructures with diameters ∼1 nm and lengths from 100 nm up to several micrometers.^40^ Depending on the roll-up vector, the SWCNTs have different diameters and different geometries, where the semiconducting (chiral) species of SWCNTs reveal fluorescence in the near-infrared (NIR) spectral wavelength range (900–1400 nm), which overlaps with the transparency window of biological tissue.^41,42^ The long fibrillary and flexible nanotubes show structural similarity to the fibrous peptide self-assemblies forming the hydrogels. Therefore, we consider them an ideal nanomaterial for the integration into peptide hydrogels as optical probes. SWCNTs are highly hydrophobic and must be individually suspended in water to reveal their characteristic fluorescence in the NIR spectral region.^43^ Several classes of dispersants are reported to suspend SWCNTs in water, e.g., surfactants,^44,45^ single-stranded DNA,^46,47^ amphiphilic polymers,^48−51^ and also proteins, peptoids, and peptides.^52−58^ Several studies showed that (Fmoc-)conjugated amino acids and peptides are able to disperse SWCNTs in an aqueous environment,^59,60^ and molecular dynamics simulation showed the hybridization of amino acid crystals with carbon nanotubes.^61,62^ Further, SWCNTs have proven high biocompatibility in several *in vivo* applications, rendering them favorable for usage in implantable hydrogels.^63−65^

The NIR-fluorescence emission of SWCNTs depends on their close environment.^66,67^ Changes in their close proximity, e.g., by analyte binding, are translated into changes in their fluorescence properties, i.e., emission wavelength shifts or fluorescence intensity changes.^47,54,67−71^ Thus, SWCNTs can be utilized as NIR-fluorescence sensors that do not suffer from photobleaching or blinking, allowing for long-term measurements.^63,72−75^ Successful integration of SWCNTs into hydrogels while taking advantage of their fluorescence properties as analyte-specific sensors has been shown.^76,77^ Several studies also demonstrated the feasibility of SWCNTs integrated into hydrogels as implantable sensor platforms^78,79^ for NIR-imaging and spectroscopy in tissue.^29,80−83^

SWCNTs encapsulated within amino acid or peptide hydrogels found a plethora of applications^84^ as functional elements to induce conductivity^8,85^ or light-triggered drug release.^86^ Further, SWCNTs were utilized as structural elements to improve the self-healing of hydrogels,^62,87^ enhance cell growth in hydrogel matrices,^5^ or alter the mechanical properties of the hydrogels.^62,88−90^ In all these applications, the SWCNTs were used either in their oxidized form, in which they lose their fluorescence properties, or their NIR-fluorescence was not exploited.

Here, we show that SWCNTs can be directly suspended by FmocFF and can be integrated into FmocFF and FmocFF/polymer hybrid hydrogels as optical probes, revealing real-time information about structural changes inside the gels and functioning as nonphotobleaching staining for the structural properties of the hydrogels. The polymers added to the FmocFF hydrogels are dextran, polyethylene glycol, and sodium alginate, chosen as additives owing to their wide usage in peptide hydrogels with biomedical applications.^9,23,91^ We follow the real-time fluorescence signal modulations upon gel formation and compare them to the kinetic changes of the storage and loss moduli measured via rheology. Compared to rheometric measurements, our fluorescent nanoprobes show a higher sensitivity to small changes inside the self-assembled hydrogels. We further show that fluorescence imaging of the SWCNTs inside the hydrogels reveals morphological characteristics of the FmocFF and FmocFF/polymer hydrogels, otherwise inaccessible using light microscopy. Additionally, we measure real-time structural changes in the FmocFF and FmocFF/polymer hydrogels upon the addition of Ca^2+^-ions via the SWCNT fluorescence, further confirming that we can follow the fate of the hydrogels in a noninvasive way. Our results open new avenues for real-time, long-term monitoring of the structure and morphology of peptide hydrogels in the NIR spectral window, either via fluorescence spectroscopy or fluorescence imaging, with numerous applications both as *in vitro* or *in vivo* bioinspired self-assembled matrices.

Since FmocFF forms the molecular matrix of our peptide hydrogels (Figure 1a), we chose to suspend SWCNT with FmocFF directly. The suspension was achieved via tip sonication of the SWCNTs in FmocFF dissolved in water with the addition of NaOH. Figure 1b shows the absorption spectrum of SWCNT@FmocFF, revealing distinct absorption peaks of the different SWCNT chiralities abundant in the sample, confirming a stable colloidal suspension of the SWCNTs. The gelation process of the peptide hydrogels with integrated SWCNTs was initiated by the solvent switch method (Figure 1c).^29^ To this end, we diluted the SWCNT@FmocFF in water to the desired final concentration in the hydrogel and added the suspension to FmocFF dissolved in dimethyl sulfoxide (DMSO). The final concentration of FmocFF in the hydrogel was 10 mM. Figure 1d shows the successful formation of self-supporting FmocFF-hydrogels with and without SWCNTs. As we aim to add the SWCNTs as fluorescent probes for single particle imaging inside the gels, we used a relatively low concentration of SWCNT@FmocFF of 0.5 mg L^–1^. Nevertheless, higher concentrations of SWCNT@FmocFF (20 mg L^–1^) could also be integrated to form self-supporting hydrogels (Figure 1d), but these gels appeared to be more viscous than gels with lower SWCNT concentrations, showing that the SWCNTs can influence the structures of the hydrogels. To rule out leakage of SWCNTs from the gels, we hydrated the gels shown in Figure 1d with 1 mL of water and measured the absorption spectra of the water after one night of incubation. We observed leakage of DMSO and FmocFF, but SWCNT-leakage could not be observed (Figure S1). Figure 1e shows the excellent fluorescence emission of the particles in the NIR spectral region. Immediately after the addition of SWCNT@FmocFF to FmocFF in DMSO and the start of the self-assembly process, we observe an increase in SWCNT@FmocFF fluorescence intensity of up to 6-fold compared to the SWCNT@FmocFF in water. We do not observe an increase in the absorption properties of the SWCNT chiralities after gelation was induced (Figure S2). Thus, we conclude that the fluorescence increase stems from the addition and accumulation of FmocFF around or in close proximity to the suspended SWCNTs. The normalized fluorescence map shows bright fluorescence peaks for all the chiralities abundant in the SWCNT@FmocFF sample (Figure 1f). For our experiments, we chose to excite the SWCNTs with an excitation wavelength of λ_*ex*_ = 730 nm, to monitor the fluorescence emission of the (10,2) chirality (λ_*em*_ ≈ 1080 nm), the (9,4) chirality (λ_*em*_ ≈ 1150 nm), and the (8,6) chirality (λ_*em*_ ≈ 1200 nm).

**Figure 1:**
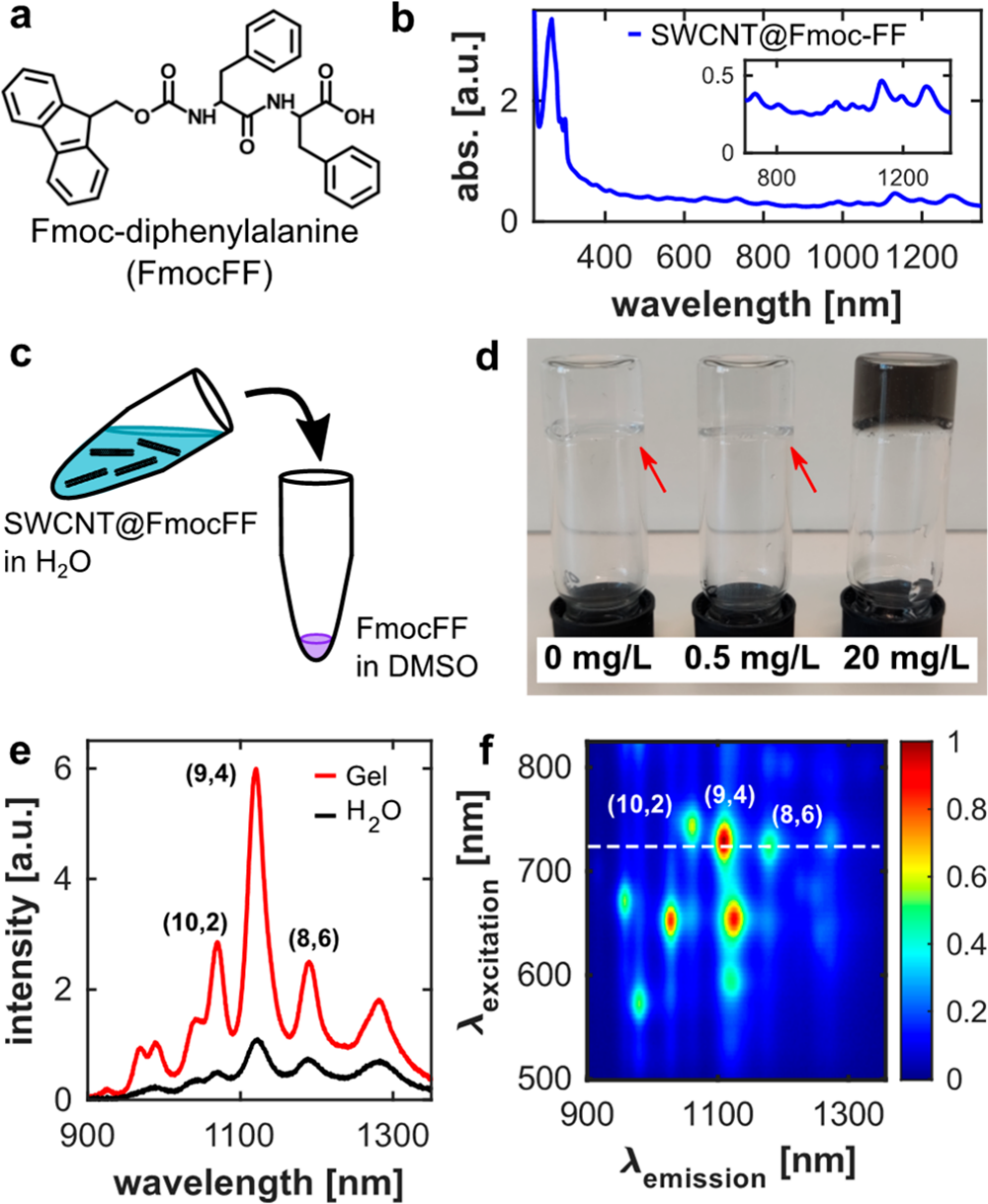
FmocFF-SWCNTs suspension and integration into peptide hydrogels. (a) Chemical structure of Fmoc-diphenylalanine (FmocFF). (b) Absorption spectra of SWCNTs suspended by FmocFF in water. Inset: NIR-absorption of the SWCNTs. (c) Schematic representation of the hydrogel formation via a solvent switch. The SWCNT@FmocFF suspension is diluted in water to the desired final concentration within the hydrogel and added to a small volume of FmocFF in DMSO to a final FmocFF concentration of 10 mM. The volume ratio of H_2_O/DMSO is 25:1. (d) Self-supported hydrogels formed with different concentrations of SWCNTs: 0 mg L^–1^ (left); 0.5 mg L^–1^ (middle), and 20 mg L^–1^ (right). Arrows indicate the transparent hydrogels. (e) Fluorescence emission spectra of the SWCNT@FmocFF suspended in water at a concentration of 0.5 mg L^–1^ (black) and in the same concentration after integration into the self-assembled FmocFF-hydrogels (red). Both spectra were measured at an excitation wavelength of λ_*ex*_ = 730 nm and an exposure time of *t*_*ex*_ = 5 s. (f) Normalized fluorescence excitation–emission map of the SWCNT@FmocFF after integration into the hydrogels, showing the fluorescence emission of all chiralities abundant in the sample. The dashed line shows the excitation wavelength of λ_*ex*_ = 730 nm, exciting the (10,2), (9,4), and (8,6) chiralities.

Carbon nanomaterials were reported as structural elements integrated into peptide hydrogels.^21,62,90^ However, we aim to implement the SWCNTs into the hydrogels as fluorescence probes for the structural properties of the FmocFF hydrogels rather than as structural support. Thus, we kept the SWCNT concentration low (0.5 mg L^–1^) in order to be able to resolve single particle fluorescence, while ensuring sufficient fluorescence intensity for the spectroscopic analysis. To assess the effect of SWCNTs on the hydrogels, we measured their mechanical properties with and without the SWCNTs. Figure 2a,b shows no influence of the SWCNTs in the applied concentration on the hydrogel formation kinetics measured by their storage and loss moduli (*G*′ and *G*′′) during hydrogelation and on the resistance of the gels to an applied strain. We also performed a creep-recovery test on the hydrogels, where we observed a similar recovery for both gels of 108% without SWCNTs and 160% with SWCNTs (Figure 2c).

**Figure 2:**
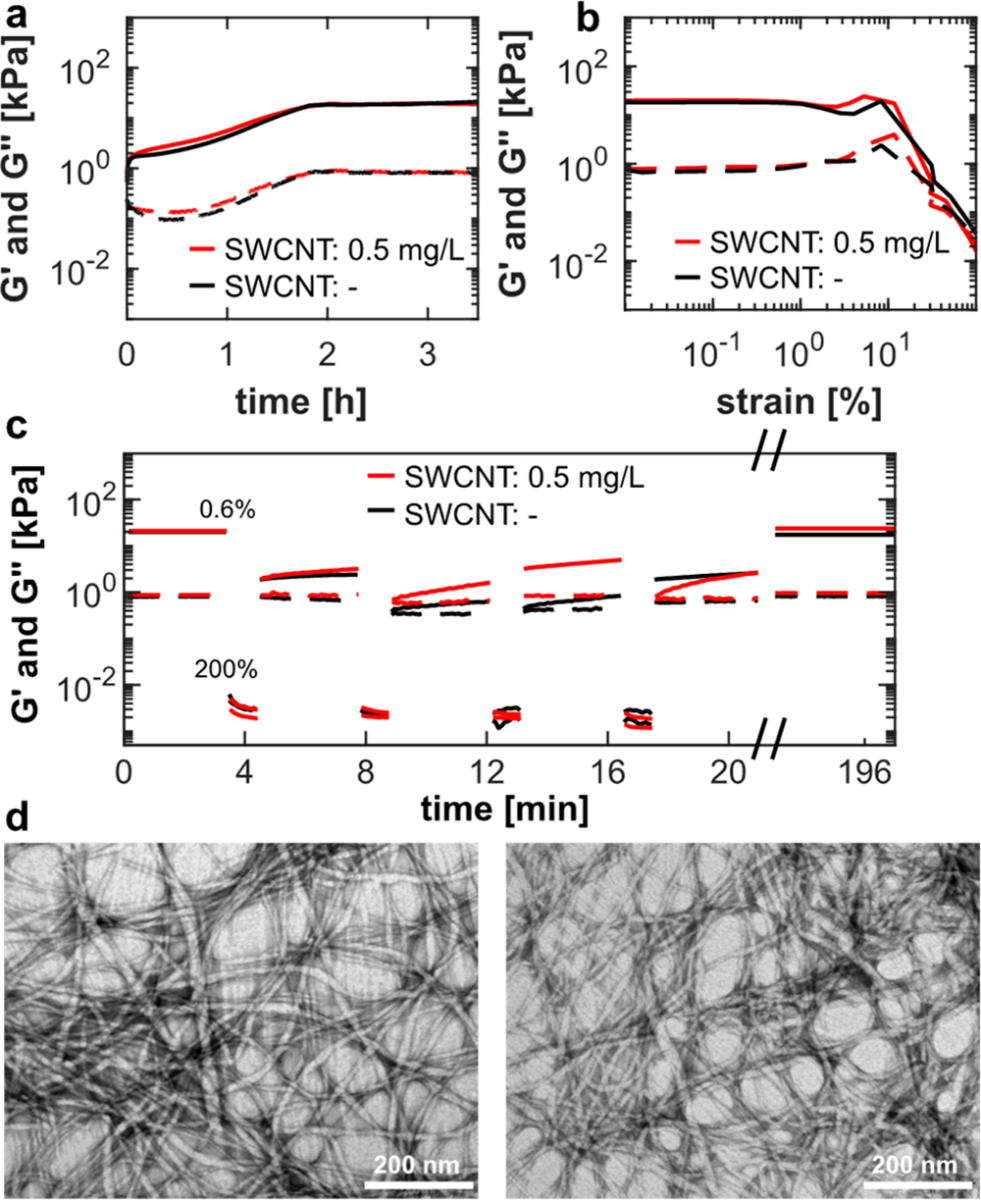
SWCNTs in the applied concentration do not affect the mechanical properties nor the morphologic structure of the hydrogels. (a) Time-dependent changes of storage (*G*′, solid lines) and loss moduli (*G*′′, dashed lines) during the peptide self-assembly with SWCNTs at a concentration of 0.5 mg L^–1^ (red) and without SWCNTs (black). Measurements were performed at a frequency of 1 Hz and a strain of 0.6%, which were determined to be in the linear viscoelastic region of the hydrogels. (b) Strain-sweep of the hydrogels with 0.5 mg L^–1^ (red) and without SWCNTs (black). (c) Creep-recovery test of the hydrogels with 0.5 mg L^–1^ (red) and without SWCNTs (black). Cycles of 0.6% and 200% strain were applied, and the recovery of storage (*G*′, solid lines) and loss moduli (*G*′′, dashed lines) were measured. After the last cycle of 200% strain, the hydrogels were measured following an equilibration time of 3 h. (d) TEM images of hydrogels without SWCNTs (left) and with 0.5 mg L^–1^ SWCNTs (right) after negative staining with uranyl acetate. Scale bars indicate 200 nm.

Further, we imaged the hydrogels via transmission electron microscopy (TEM) to observe their morphological structure (Figure 2d). TEM images of the gels with and without SWCNTs show similar morphologies of long fibrous, entangled peptide assemblies for both of the gels, in accordance with previous studies.^13^ These results prove that the SWCNTs in low concentrations do not affect the mechanical properties nor the morphological structure of the hydrogels, thus, they can be utilized primarily as optical probes.

Many studies implement additives, such as polymers, into peptide hydrogels to tune their mechanical and structural properties for different applications.^1,23,26,91^ We therefore chose to extend our hydrogel-encapsulated SWCNT platform to FmocFF/polymer hybrid hydrogels, adding dextran (Dex), polyethylene glycol (PEG), or sodium alginate (SA).^9,22,91^ The mechanical properties and the kinetics of the gelation process for the FmocFF/polymer hybrid hydrogels measured by rheometry are not affected by the addition of the SWCNTs (Figure 3a). Based on the observed changes in storage (*G*′) and loss (*G*′′) moduli, the gelation process equilibrates after ca. 2 h in the case of FmocFF, FmocFF/Dex, and FmocFF/PEG and after ca. 3 h in the case of FmocFF/SA. The fluorescence emission intensity and wavelength of the SWCNTs depend on the dielectric environment in the close proximity of the SWCNT surface,^39^ which can change by the accumulation of organic molecules on the SWCNT surface and the replacement of water molecules. The extent of the fluorescence changes is often reported to be dependent on the SWCNT chirality, due to differences in the surface structure and the curvature of SWNCTs.^46,54,92,93^ Thus, we expect the addition and self-assembly of FmocFF in the presence of SWCNTs to affect their fluorescence emission. Indeed, we see a sharp, rapid increase in the SWCNT fluorescence for all the hydrogels immediately after the solvent switch, due to the addition of FmocFF, followed by a gradual decrease in fluorescence emission intensity with the beginning of the peptide self-assembly (Figure S3).

**Figure 3:**
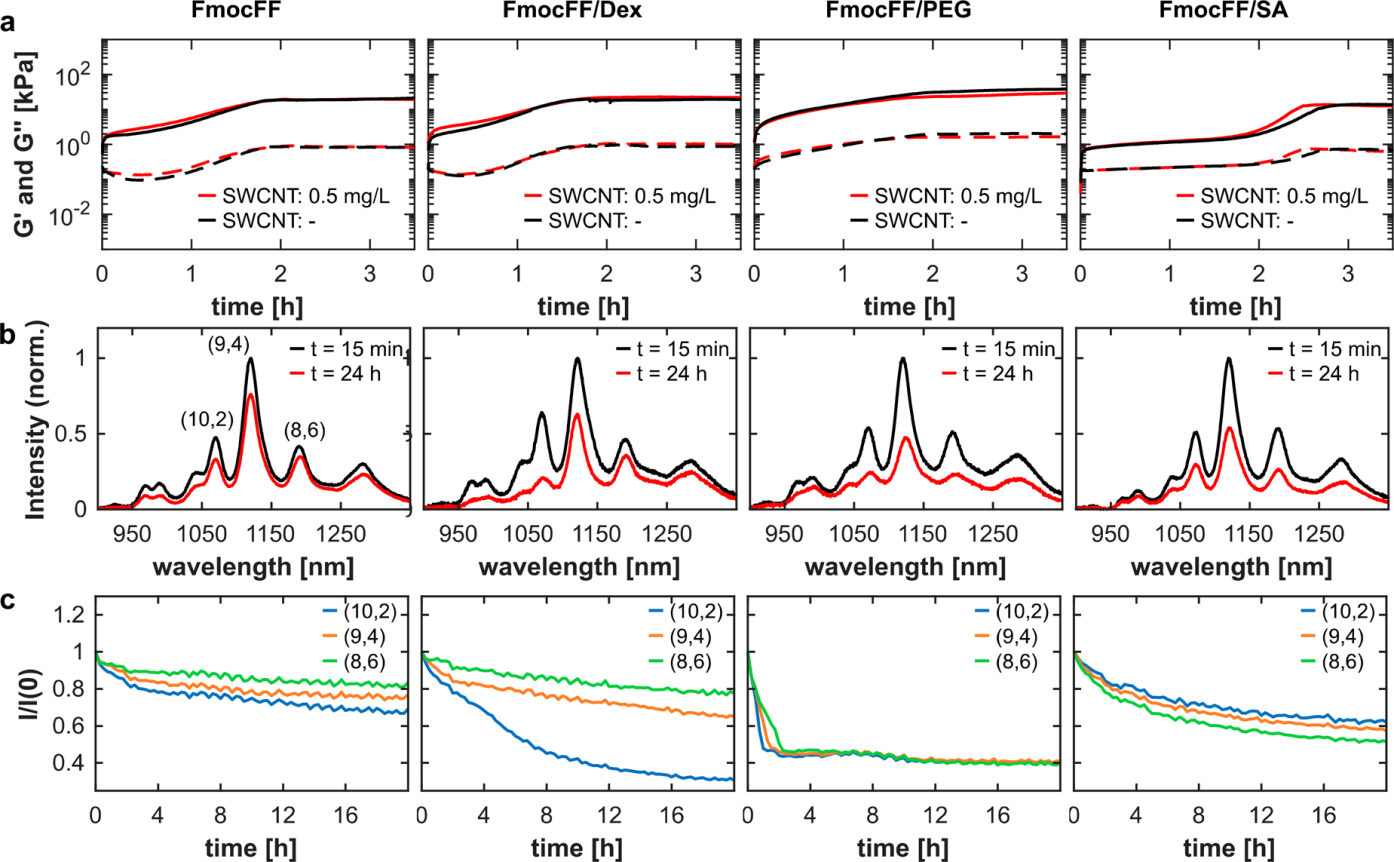
Rheology and fluorescence spectroscopy of the hydrogelation of FmocFF and FmocFF/polymer hydrogels. (a) Time-dependent changes of the storage (*G*′, solid lines) and loss moduli (*G*′′, dashed lines) during hydrogel formation with SWCNTs at a concentration of 0.5 mg L^–1^ (red) and without SWCNTs (black). (b) Normalized fluorescence emission spectra of SWCNTs integrated into the hydrogels, 15 min after the solvent switch (black) and after 24 h of gelation time (red) at an excitation wavelength of λ_*ex*_ = 730 nm. (c) Time-dependent fluorescence intensity changes of three different SWCNT chiralities indicated in (b): blue, (10,2); orange, (9,4); green, (8,6). All measurements were performed in triplicates.

Comparing the spectra of the SWCNTs in the beginning and after 24 h of the self-assembling process shows a chirality-dependent significant decrease of fluorescence emission in all four hydrogels (Figure 3b) with negligible changes in the absorption (Figure S4). Following the time-dependent fluorescence emission decrease over 20 h for the three main chiralities excited at λ_*ex*_ = 730 nm, we observe a fluorescence intensity decrease, which is highly dependent on the composition of the hydrogels and, especially in the case of FmocFF/Dex, is highly dependent on the SWCNT chirality (Figure 3c). Additionally, the intensity changes are accompanied by wavelength shifts of the fluorescence peak emission, as expected upon changes in the density of molecules on the surface of the SWCNTs due to FmocFF self-assembly (Figure S5). The intensity modulations and wavelength shifts over time stem from the changes in the dielectric environment around the SWCNT surface.^67,94^ These changes are induced by the rearrangement and self-assembly of molecules, polymer chains, and FmocFF close to the SWCNT surface. Moreover, spectral red-shifts of SWCNT fluorescence are often attributed to a higher molecular density of the SWCNT corona phase. Any fluorescence modulation is highly dependent on the specific configuration of the corona phase and solvent molecules around the SWCNT surface, as manifested in the large variability of the intensity and wavelength responses in the different self-assembled hydrogels. The SWCNT fluorescence in FmocFF/PEG hydrogels shows the largest wavelength shift of 4–6 nm, compared to FmocFF, FmocFF/Dex, and FmocFF/SA, where we observe less than a 2 nm shift for all chiralities. The fluorescence intensity response of the SWCNTs in FmocFF and in FmocFF/Dex is very similar except for the (10,2) chirality in FmocFF/Dex, which shows a higher intensity decrease. In FmocFF/PEG, the SWCNT fluorescence shows a high-intensity decrease of ca. 60%, which is accompanied by the largest wavelength shift described above, while in FmocFF/SA, we observe an intensity decrease of 40%, which is accompanied by only a minor wavelength shift of <1 nm. Therefore, we hypothesize that the arrangement of PEG polymers in the close proximity of the SWCNTs causes a bigger change in the dielectric environment than the polysaccharides sodium alginate and dextran, resulting in a larger wavelength shift to the SWCNT fluorescence.

Previous studies have reported chirality-dependent response of the SWCNT fluorescence to changes in the SWCNT corona phase.^92,95,96^ Each SWCNT chirality is characterized by its diameter and curvature, which affects the interaction with FmocFF and the polymer around it, e.g., in density or 3D conformation. These differences can lead to a higher or lower fluorescence response of the respective chiralities.

The intensity changes for all hydrogels are remarkably slower compared to the self-assembly kinetics observed in the rheometric measurements. We attribute this effect to the high sensitivity of SWCNTs to small changes in their close environment.^97^ Microscopic changes might not affect the mechanical stability of the hydrogels on a macroscopic scale as measured by the rheometer, but they are still important for a complete equilibration of the peptide hydrogels. In fact, many studies on peptide hydrogels report aging times for their materials for up to 3 days.^22,36^

To visualize the SWCNTs inside the hydrogels and capture their morphology during the gelation process, we imaged the hydrogels before complete equilibration after 1 h of gelation time. In Figure 4, we show bright-field images of the hydrogels and the respective NIR-fluorescence channel images of the SWCNTs integrated within the hydrogels. Supporting Videos S1–S4 show the diffusion of the SWCNTs inside the hydrogels measured over 5 s with an exposure time of 100 ms. We determined the mean square displacement (MSD) over *t* = 100 ms of the SWCNTs inside the hydrogels (Table S1). For all four samples, we see individually dispersed, fluorescent SWCNTs, where the distribution and the MSD of the particles inside the hydrogels strongly depend on their polymer additive. In the case of FmocFF and FmocFF/Dex, bright-field images reveal circular nucleation sites of the hydrogels, corresponding to areas without SWCNTs presence in the NIR channel, which appear as circular darker regions. These regions may appear to have some unique radial features in the bright-field channel that are not present in other areas of the image, although they are difficult to identify due to the low contrast. Moreover, we observe SWCNTs diffusing inside the FmocFF (MSD_*FmocFF*_ = 0.152 μm^2^) and FmocFF/Dex (MSD_*FmocFF/Dex*_ = 0.245 μm^2^) hydrogels, but only in their confined regions (Supporting Videos S1 and S2). FmocFF/PEG gels do not show such confinement of particles, but rather random distribution of the SWCNTs with a higher MSD_*FmocFF/PEG*_ = 0.758 μm^2^ (Supporting Video S3). Accordingly, circular nucleation sites cannot be identified in the bright-field images of FmocFF/PEG. FmocFF/SA show thick fibrillary structures (Figure 4), and also circular nucleation sites (Figure S6), with much higher contrast, compared to the other gels. The SWCNTs show very little to no diffusion inside the hydrogel matrix (MSD_*FmocFF/SA*_ = 0.014 μm^2^), as they seem to be bound to the fibers of the FmocFF/SA hydrogels (Figure 4 and Supporting Video S4). For all FmocFF/polymer hydrogels, we can observe similar structural properties in the hydrogels without SWCNTs, proving that the morphology of the hydrogels is not affected by SWCNT addition (Figure S7).

**Figure 4:**
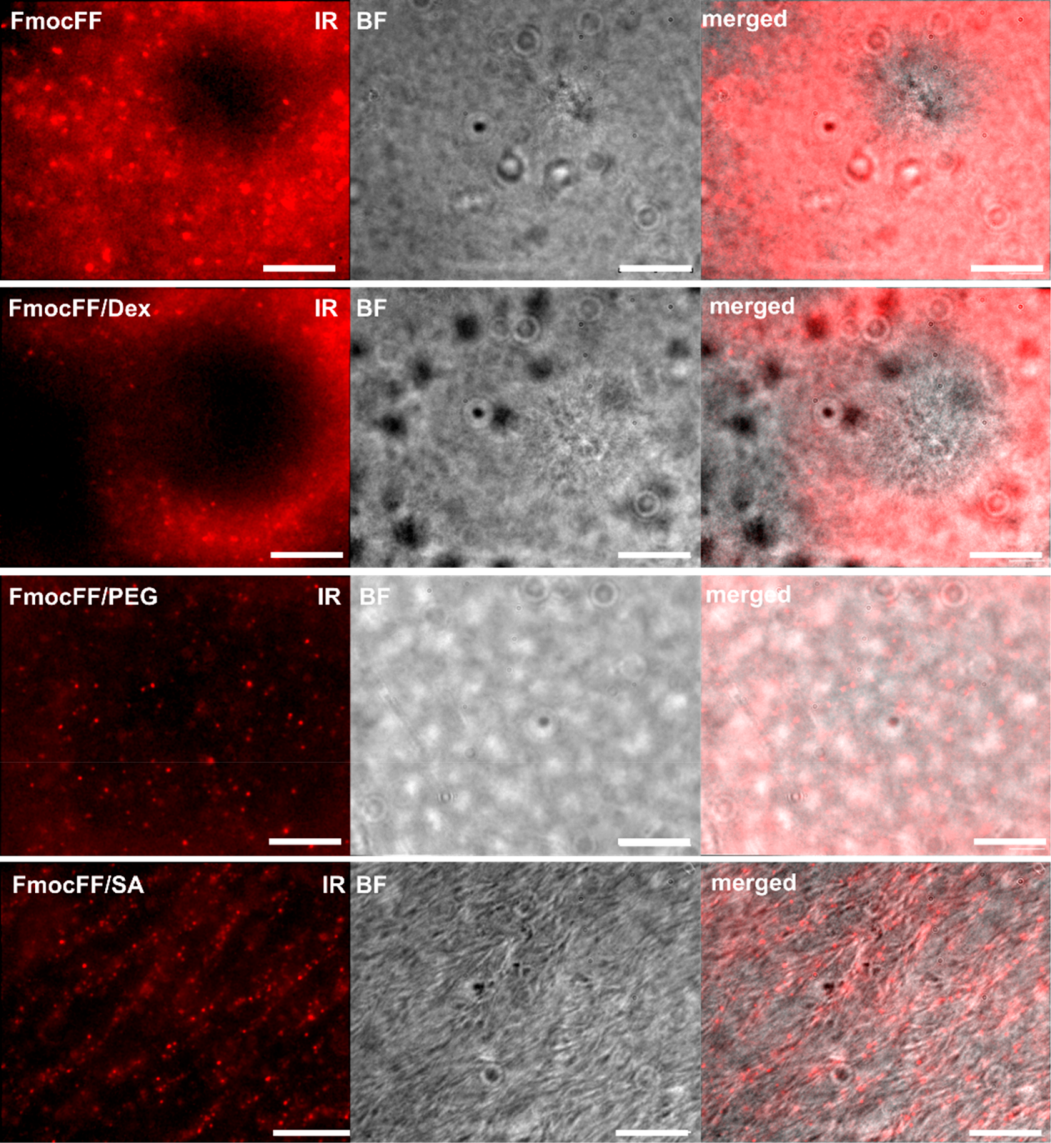
Fluorescence NIR-imaging of SWCNTs inside FmocFF and FmocFF/polymer hydrogels. Images of the NIR fluorescence channel (IR), the respective bright-field image (BF), and the merged brightfield and IR-channel of the four SWCNT-encapsulating peptide hydrogels after 1 h of gelation time. Scale bar indicates 20 μm.

The NIR fluorescence images of the SWCNTs within the hydrogels (Figure 4) demonstrate that SWCNTs can be used to “stain” the hydrogels and track their morphology. As the bright-field images of the hydrogels have very low contrast, the SWCNTs allow for the identification of the nucleation sites in FmocFF and FmocFF/Dex gels, which correspond to the circular darker regions in the NIR images with low fluorescence intensity, and highlight the fibrillary structure in the case of FmocFF/SA. The time span required for the SWCNTs to settle down and be fully integrated within the hydrogel matrix correlates to the slow kinetics of the fluorescence changes of the SWCNTs measured by spectroscopy during the gelation (Figure 3c). Thus, the SWCNT motion, together with spectral changes in their fluorescence emission, serves as an indicator for the full equilibration of the self-assembling process of peptide/polymer hydrogels and to rationalize their aging time.

After a gelation time of 5 h, diffusion of the SWCNTs can still be observed inside the gels, although the rheologic measurements show that the mechanical properties of the gels are stable at this time point (Supporting Videos S5–S8). After 24 h of gelation time, the particles do not show any diffusion inside the gel, and they appear to be completely immobilized (Supporting Videos S9–S12). Still, the characteristic distribution pattern of the SWCNTs inside the FmocFF/polymer hydrogels described above is maintained (Figures S8 and S9).

The addition of Ca^2+^-ions can induce hydrogelation in aqueous FmocFF-solutions,^98^ affecting the rheological properties of FmocFF-hydrogels.^1,99^ Further Ca^2+^ acts as a strong cross-linker between sodium alginate polymer strands, while cross-linking between PEG or dextran-strands is not reported. Due to these effects, we expect to induce structural changes in the FmocFF/polymer hydrogel matrices upon the addition of Ca^2+^-ions, resulting in a change in their mechanical properties. Since we showed that the self-assembly of the FmocFF and the rearrangement of the polymers inside the hydrogel-matrix is translated to a fluorescence signal (Figure 3c), we also expected the addition of Ca^2+^-ions to induce a change in the fluorescence signal of the SWCNTs inside the hydrogels, whenever it induces a structural change (Figure 5a). Measuring the fluorescence intensity changes of the SWCNTs upon the addition of Ca^2+^ (1 mM) to the equilibrated hydrogels, we indeed observed a strong decrease in fluorescence intensity in the case of FmocFF, FmocFF/Dex, and FmocFF/SA, whereas FmocFF/PEG does not show a significant change in its fluorescence emission (Figure 5b). To exclude fluorescence quenching effects of Ca^2+^ on SWCNTs, we incubated sodium cholate or sodium dodecyl sulfate suspended SWCNTs at a concentration of 0.5 mg L^–1^ with Ca^2+^ (1 mM), but no fluorescence quenching was observed (Figure S10). Similar to the initial gelation process, we can see chirality-dependent fluorescence response of the SWCNT chiralities in FmocFF/Dex (Figures 5b and S11). The same effect can be observed for the fluorescence emission wavelength, which shifts in the case of FmocFF, FmocFF/Dex (except for the (10,2) chirality), and FmocFF/SA, but remain invariant for FmocFF/PEG (Figure S11). Again, we attribute a change in the emission wavelength to a change in the density of molecules in the close surrounding of the SWCNT surface induced by the addition and cross-linking of FmocFF molecules or polymer-chains. In the case of FmocFF/SA, we can observe significantly slower kinetics compared to FmocFF and FmocFF/Dex. We assume that the cross-linking process between the SA chains is slower than the coordination and cross-linking of the self-assembled peptide fibers. Measuring the relative change of the storage and loss moduli of the FmocFF and FmocFF/polymer hydrogels after the addition of Ca^2+^, confirms our observations. FmocFF, FmocFF/Dex, and FmocFF/SA show an increase in their storage and loss moduli, while FmocFF/PEG shows no significant change in *G*′ and a small decrease in *G*′′ (Figures 5c and S12). Dextran is not reported to complex Ca^2+^-ions, and thus we expect FmocFF and FmocFF/Dex hydrogels to show a similar behavior toward the addition of ions. PEG is reported to complex Ca^2+^-ions through neighboring oxygen atoms in the PEG chain, while hydrogelation is not reported to be induced.^100^ We hypothesize that the PEG chains in the gel complex the Ca^2+^-ions, so they are less abundant for structural changes in the FmocFF assemblies. The structural changes induced through the complexation by PEG seem to be insufficient to induce significant changes in the mechanical properties of the hydrogels (Figure 5c). To further show the potential of SWCNTs as photostable fluorescence sensors in the hydrogels, we performed the Ca^2+^ addition on hydrogels that were left to age for 1 week, showing comparable results (Figure S13). We conclude that SWCNTs integrated into FmocFF/polymer hydrogels can report on structural changes inside the hydrogel matrix and reveal their kinetics.

**Figure 5:**
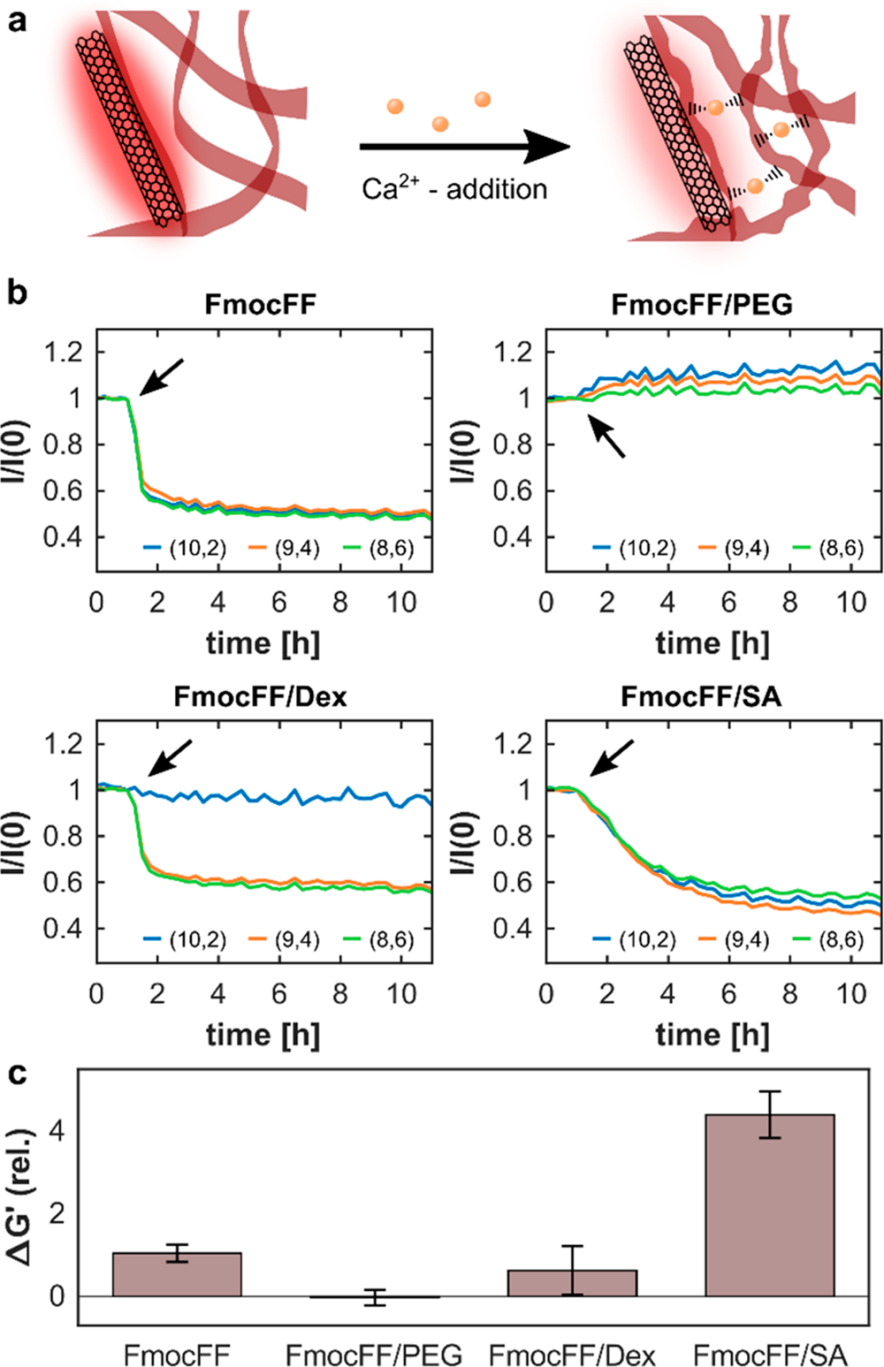
Time-dependent fluorescence response of SWCNTs to structural changes in FmocFF and FmocFF/polymer. (a) Structural changes inside the hydrogel matrix induced by the addition of Ca^2+^-ions give rise to modulations in the fluorescence emission. (b) Normalized time-dependent fluorescence intensity changes of the different SWCNT chiralities after the addition of 1 mM Ca^2+^: blue, (10,2); orange, (9,4); green, (8,6). Arrows indicate the addition of Ca^2+^. All measurements were performed in triplicates. (c) Changes in storage modulus after the addition of 1 mM Ca^2+^ to the equilibrated hydrogels. Error bars show the mean and standard deviation (*n* = 3).

In this study, we demonstrated that NIR-fluorescent SWCNTs could be directly suspended by FmocFF and integrated into FmocFF and FmocFF/polymer hydrogels without affecting their mechanical or morphological properties. We proved that the integrated SWCNTs could serve as NIR optical probes that translate structural changes in their close surrounding into changes in their fluorescence signal. In the case of FmocFF/Dex, we observed a chirality-dependent fluorescence response, which can be exploited in future studies with chirality-pure SWCNT samples, shown in Figure S14 with a (6,5)-chirality enriched SWCNT sample. Since the SWCNTs are sensitive to nanoscopic changes in their close proximity, they can transduce local changes within the hydrogel to fluorescence modulation, showing an unequivocal advantage to rheologic measurements which cannot capture these microscopic changes. The aging time of the hydrogels can be determined more precisely, and as demonstrated following the addition of Ca^2+^, any structural changes can be followed in real-time and in a noninvasive way.

Fluorescence imaging of the SWCNTs inside the hydrogels can reveal the characteristic morphological structure of the gels, e.g., circular nucleation sites which correspond to darker circular regions in the NIR fluorescence channel. The SWCNTs serve as NIR fluorescent staining that does not suffer from photobleaching nor blinking, allowing long-term imaging of the hydrogels. These optical probes enable us to follow the formation of the hydrogels over time and, e.g., in the case of FmocFF and FmocFF/Dex, highlight the nucleation sites with a much better contrast compared to the bright-field images.

In conclusion, we showed that SWCNTs integrated into self-assembled peptide hydrogels are versatile tools for imaging and spectroscopy, allowing for real-time monitoring of processes inside the gels. The synthesis and application of peptide hydrogels are, however, not limited to FmocFF-peptides but extended to a library of Fmoc-conjugated peptides. As the Fmoc-moiety stabilizes the SWCNTs, we predict that the application of the SWCNTs inside the gels can be extended to other Fmoc-peptide gelators. As peptide hydrogels are often used as injectable or implantable hydrogels, SWCNTs open up numerous opportunities for biomedical application due to their fluorescence in the NIR transparency window of biological tissue and their lack of photobleaching.

## Supporting Information Available

The following files are available free of charge. Supporting Information (PDF) Supporting Video 1–12 (AVI) The Supporting Information is available free of charge at https://pubs.acs.org/doi/10.1021/acs.nanolett.2c01587.

Experimental section; absorption spectra of SWCNTs in water and in the hydrogels; SWCNTs leakage from gels; fluorescence intensity changes of SWCNTs directly after solvent-switch; time-dependent wavelength shifts of SWCNTs inside the hydrogels during hydrogelation; displacement of the SWCNTs inside the gels; fluorescence and bright-field microscopy images of hydrogels with and without SWCNTs; Quenching of SWCNTs with Ca^2+^; fluorescence spectra of SWCNTs inside the hydrogels before and after incubation with Ca^2+^; time-dependent wavelength shifts of SWCNTs inside the hydrogels during incubation with Ca^2+^; Ca^2+^ addition to hydrogels after 1 week of aging time; experiment with (6,5) enriched SWCNTs (PDF)
Video S1: SWCNTs integrated into FmocFF-1h (AVI)
Video S2: SWCNTs integrated into FmocFF-Dex-1h (AVI)
Video S3: SWCNTs integrated into FmocFF-PEG-1h (AVI)
Video S4: SWCNTs integrated into FmocFF-SA-1h (AVI)
Video S5: SWCNTs integrated into FmocFF-5h (AVI)
Video S6: SWCNTs integrated into FmocFF-Dex-5h (AVI)
Video S7: SWCNTs integrated into FmocFF-PEG-5h (AVI)
Video S8: SWCNTs integrated into FmocFF-SA-5h (AVI)
Video S9: SWCNTs integrated into FmocFF-24h (AVI)
Video S10: SWCNTs integrated into FmocFF-Dex-24h (AVI)
Video S11: SWCNTs integrated into FmocFF-PEG-24h (AVI)
Video S12: SWCNTs integrated into FmocFF-SA-24h (AVI)

## Supplementary Material

nl2c01587_si_001.pdf

nl2c01587_si_002.avi

nl2c01587_si_003.avi

nl2c01587_si_004.avi

nl2c01587_si_005.avi

nl2c01587_si_006.avi

nl2c01587_si_007.avi

nl2c01587_si_008.avi

nl2c01587_si_009.avi

nl2c01587_si_010.avi

nl2c01587_si_011.avi

nl2c01587_si_012.avi

nl2c01587_si_013.avi
