# Supplementary material for: Single-Walled Carbon Nanotubes as Fluorescent Probes for Monitoring the Self-Assembly and Morphology of Peptide/Polymer Hybrid Hydrogels
Source: Nano Lett. 2022 Oct 19;22(22):9205–14. doi: 10.1021/acs.nanolett.2c01587 (PMC9706665; doi:10.1021/acs.nanolett.2c01587)
Supplement: Supplementary file 1 — nl2c01587_si_001.pdf [file nl2c01587_si_001.pdf]

Single-walled carbon nanotubes as fluorescent probes for monitoring the self-assembly and morphology of peptide/polymer hybrid hydrogels

-Supporting Information-

Verena Wulf,[a] *and Gili Bisker* [a,b,c,d,] *

[a] Department of Biomedical Engineering, Faculty of Engineering, Tel-Aviv University, Tel Aviv 6997801, Israel

[b] The Center for Physics and Chemistry of Living Systems, Tel-Aviv University, Tel Aviv 6997801, Israel

[c] Center for Nanoscience and Nanotechnology, Tel-Aviv University, Tel Aviv 6997801, Israel

[d] Center for Light Matter Interaction, Tel-Aviv University, Tel Aviv 6997801, Israel

* bisker@tauex.tau.ac.il

Experimental Section

Suspension of single-walled carbon nanotubes with Fluorenylmethyloxycarbonyl–diphenylalanine (FmocFF): FmocFF (Bachem) was dissolved in water in a concentration of 5 mg mL^{-1} with the addition of NaOH (0.5 m, ca. 50 µL on 2 mL FmocFF solution) until FmocFF was dissolved at a pH of 9-10. 750 µL of FmocFF solution was added to 1 mg HiPCO-SWCNT (NanoIntegris) and was bath sonicated for 10 min followed by tip sonicated for 2 x 20 min on ice (QSonica, Q125 sonicator, 12 W). After sonication, 750 µL of water was added to decrease the pH. No gelation was observed in the nanotube suspension. To remove nanotube bundles and impurities, the resulting suspension was centrifuged two times for 90 min at 20,000 g, and 80% of the supernatant was kept after each centrifugation step. The concentration of the SWCNT@FmocFF suspension was determined spectroscopically with an extinction coefficient of $\varepsilon_{632\ nm} = 0.036\ L \cdot mg^{-1} \cdot cm^{-1}$ and was found to be 200-300 mg L^{-1}, depending on the batch.

Hydrogel preparation *via* 'solvent-switch' method and Ca^{2+}-addition: For the hydrogel formation, a solution of FmocFF in DMSO (250 mM) was diluted with water at a ratio of 1:25 to obtain a final FmocFF concentration of 10 mM in the hydrogel. For the hybrid hydrogels, the added water contained 0.5 mg L^{-1}SWCNTs and the respective concentration of sodium alginate (sodium alginate from brown algae, Sigma), polyethylene glycol (Santa Cruz Biotechnology), or dextran (dextran from *leuconostoc ssp.*, Sigma). A solvent switch was performed in an Eppendorf tube, and the solution was immediately transferred into a well-plate for spectroscopy (200 µL). After a gelation time of 10-15 h, 50 µL of water were added on top of the hydrogels to hydrate them and prevent them from drying out.

For fluorescence imaging of the SWCNTs inside the gels, 50 µL of the solution was drop cast on a microscope slide and covered with a coverslip resulting in a gel with ca. 20 µm thickness. The fluorescence response to $CaCl_2$ addition was measured by adding 60 µL of $CaCl_2$ solution to the hydrogels in the well-plate to a final concentration of 1 mM $CaCl_2$.

Rheometric measurements: Rheological properties of the hydrogels were measured using a rotational rheometer (Discovery HR2, TA Instruments, USA) with a paralleled plate geometry with a diameter of 20 mm and a temperature of 25°C, and a gap size of 1 mm. For the time sweep oscillatory measurements, a freshly prepared FmocFF solution in DMSO (250 mM) was used for each measurement. The hydrogel was prepared as described above, and a sample of 370 µL was subjected immediately on the plate. Time sweep oscillatory measurements were conducted at a frequency of 1 Hz and a strain of 0.6%. These values were found to be in the linear viscoelastic region of the hydrogels. Amplitude sweep measurements were conducted at

a strain of 0.01-100% at a frequency of 1 Hz. Creep recovery tests were performed on gels that were initially equilibrated for 3 h. Subsequently, the gels were exposed for 60 s to a strain of 200%, and gel recovery was measured at a strain of 0.6% for 200 s. After 4 cycles of strain recovery measurements, we allowed the gel to recover for 3 h, while monitoring the recovery of its storage and loss moduli. Changes in mechanical properties of the hydrogels after the addition of $CaCl_2$ were measured after equilibration of the hydrogels for 3 h. 100 µL of $CaCl_2$ solution was added to the gel to a final concentration of 1 mM $CaCl_2$. Subsequently, time sweep oscillatory measurements were conducted at a frequency of 1 Hz and a strain of 0.6% until equilibration of the hydrogel. All measurements were performed with three repetitions.

Fluorescence imaging: Images were taken *via* an inverted fluorescence microscope (Olympus IX83). Transmission bright-field images were captured under the illumination of an LED-light source and captured with an EMCCD camera (Andor, iXon Ultra) with an exposure time of t_{ex} = 200 ms. The SWCNTs fluorescence was excited by a CW-laser (MDL-MD-730-1.5W, Changchun New Industries) at an excitation wavelength of 730 nm and an excitation power of 200 mW. Laser excitation light was directed to the sample by a dichroic mirror (900 nm lp, chroma, T900lpxxrxt), and the NIR emission of the SWCNTs was detected after an additional 900 nm long-pass emission filter (chroma, ET900lp) with an InGaAs-camera (Raptor, Ninox 640 VIS-SWIR) with an exposure time of t_{ex} = 100 ms. All images were processed by ImageJ, GIMP, and MATLAB. Images were taken from three different gels, respectively.

Fluorescence spectroscopy: Fluorescence emission spectra were recorded in a 96-well-plate mounted on an inverted microscope (Olympus IX73). A super-continuum white-light laser (NKT-photonics, Super-K Extreme) with a bandwidth filter (NKT-photonics, Super-K Varia, $\Delta\lambda$ = 20 nm) was coupled into the microscope as the excitation light source. If not stated otherwise, spectra were recorded at an excitation wavelength of λ_{ex} = 730 nm with 20 mW excitation power. Fluorescence emission was spectrally resolved using a spectrograph (Spectra Pro HRS-300, Princeton Instruments) with a slit-width of 500 µm and a grating (150 g/mm). The fluorescence intensity spectrum was recorded by an InGaAs-camera (PylonIR, Teledyne Princeton Instruments) with an exposure time of t_{ex} = 5 s. All measurements were performed with three repetitions. Excitation-emission maps were recorded using an excitation wavelength range of 500-840 nm in 2 nm steps. Intensity changes and wavelength shifts were determined by fitting the peaks of the (10,2), (9,4), and (8,6)-chiralities with a Lorentzian function.

Single nanoparticle tracking: Nanotube displacement from the videos was determined using TrackMate toolbox in Fiji (ImageJ).[1]

Absorption spectroscopy: Absorption spectra were recorded in a UV-Vis-NIR spectrophotometer (Shimadzu UV-3600 Plus).

Transmission electron microscopy: 10 µl of the gel was applied to a carbon-coated grid directly after the solvent switch and incubated for 30 s. Subsequently, the grids were washed with water for 30 s, followed by staining with 10 µl of 2% (w/v) uranyl acetate for 1 min. Excess stain solution was removed by blotting on a filter paper, and the grid was left to dry. The negatively stained samples were imaged using a JEM-1400Plus TEM operating at 80 kV. Images were recorded using SIS Megaview III camera, iTEM, the TEM imaging platform (Olympus).

Additional Figures

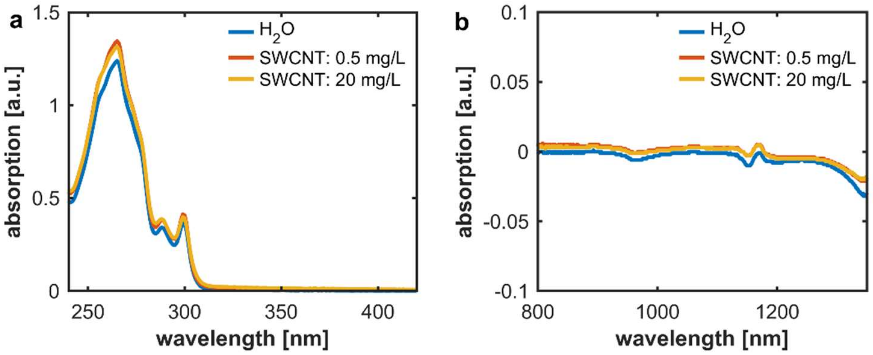

Figure S1: Leakage test of SWCNTs from FmocFF hydrogels. a) Absorption spectrum of FmocFF-leakage from FmocFF-hydrogels. b) Absorption spectrum of SWCNT-leakage from FmocFF-hydrogels.

To determine the leakage, 1 mL of water was added to the hydrogels shown in Figure 1d and incubated overnight. Subsequently, we measured the absorption spectra of the water via UV-vis-NIR absorption spectroscopy. While we can observe leakage of FmocFF from the gel (Figure S1a), we cannot observe significant SWCNT-leakage in the NIR wavelength range (Figure S1b). The absorption pattern in Figure S1b is due to DMSO leakage from the hydrogel.

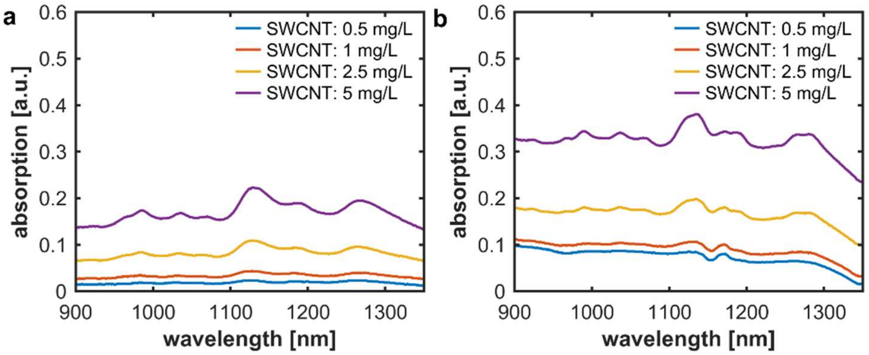

Figure S2: Absorption spectra of SWCNT@FmocFF in water and within FmocFF-hydrogels. a) NIR-absorption spectra of SWCNT@FmocFF in water at different concentrations. b) NIR-absorption spectra of SWCNT@FmocFF integrated into FmocFF-hydrogels (10 mM) at different SWCNT-concentrations. Differences in the spectra are partly due to the absorption of DMSO in the hydrogel spectra in (b).

Absorption spectra of SWCNT integrated within the gels show a higher background, but the signal-to-noise ratio of the absorption peaks is comparable to the particles in water. Therefore, the increase in SWCNT-fluorescence after solvent switch for gel formation cannot be attributed to higher absorption.

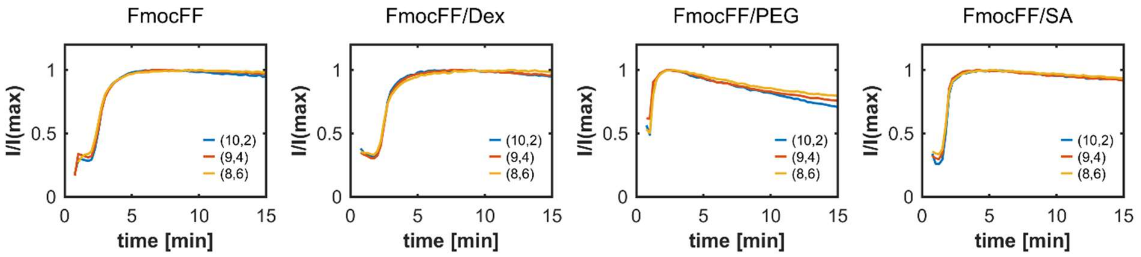

Figure S3: Fluorescence intensity changes of SWCNTs immediately after solvent switch. Normalized time-dependent fluorescence intensity changes were measured in the first 15 min of the gelation process for three different SWCNT chiralities: blue – (10,2); orange – (9,4); green – (8,6). The time-traces were measured at time intervals of Δt = 15 s during the addition of the hydrogel solution into the well-plate.

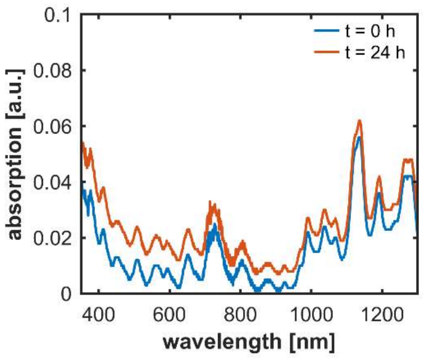

Figure S4: Absorption spectra of SWCNTs in FmocFF-hydrogels. Absorption spectra were measured at the beginning of the gelation time (blue) and after 24 h of gelation time (red). Differences in the spectra are due to changes in the absorption of the hydrogel during gelation.

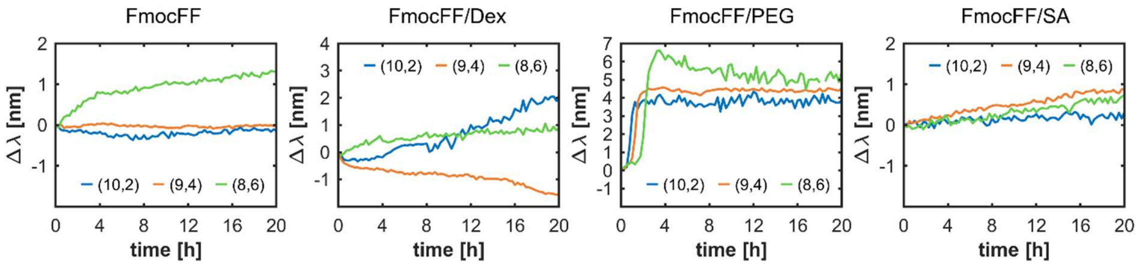

Figure S5: Fluorescence wavelength shift of SWCNTs during gelation. Time-dependent fluorescence wavelength shifts during the hydrogelation of the three different SWCNT chiralities: blue – (10,2); orange – (9,4); green – (8,6).

Table S1. Mean square displacement of SWCNTs in the hydrogels for t = 100 ms

	**1 h**	**5 h**	**24 h**
**FmocFF**	0.152 μm^2	0.226 μm^2	0.005 μm^2
**FmocFF/PEG**	0.758 μm^2	0.892 μm^2	0.002 μm^2
**FmocFF/Dex**	0.245 μm^2	0.231 μm^2	0.014 μm^2
**FmocFF/SA**	0.014 μm^2	0.020 μm^2	0.002 μm^2

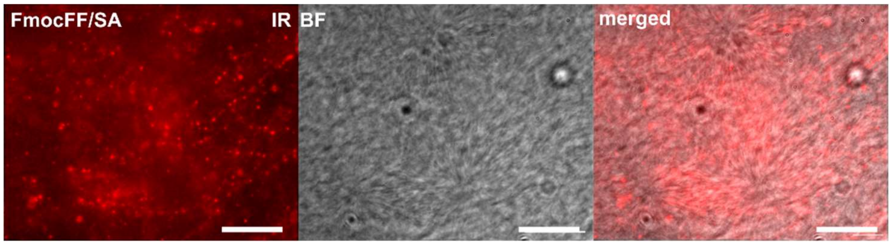

Figure S6: Nucleation sites in FmocFF/SA. Images showing examples for the NIR fluorescence channel (left), the respective bright-field image (middle) and the merged images (right) for the four hydrogels. Scale bar indicates 20 μm.

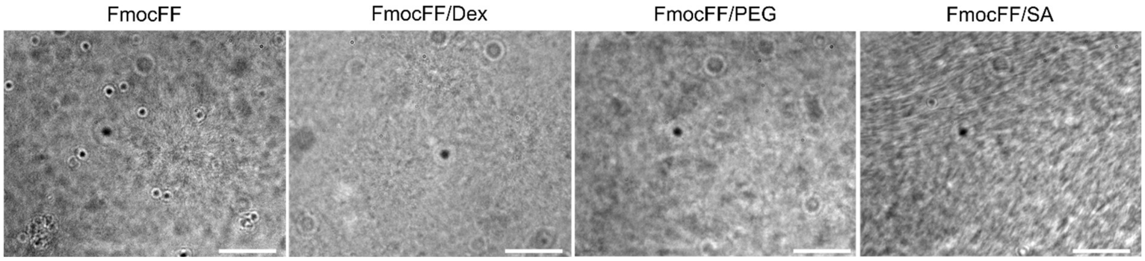

Figure S7: Bright-field images of FmocFF and FmocFF/polymer hydrogels in the absence of SWCNTs. Images showing the bright-field image for the four hydrogels. Scale bar indicates 20 μm.

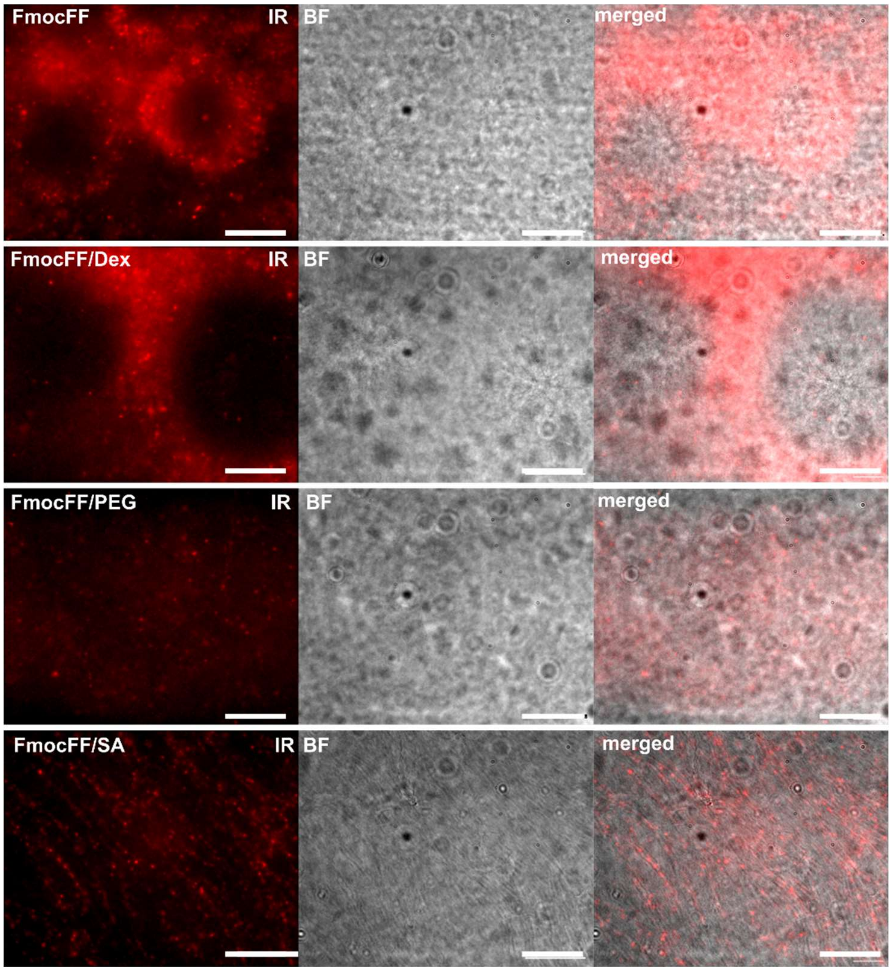

Figure S8: Fluorescence NIR-imaging of SWCNTs within FmocFF and FmocFF/polymer hydrogels after 5 h gelation time. Images showing examples for the NIR fluorescence channel (left), the respective bright-field image (middle), and the merged images (right) for the four hydrogels. Scale bar indicates 20 µm.

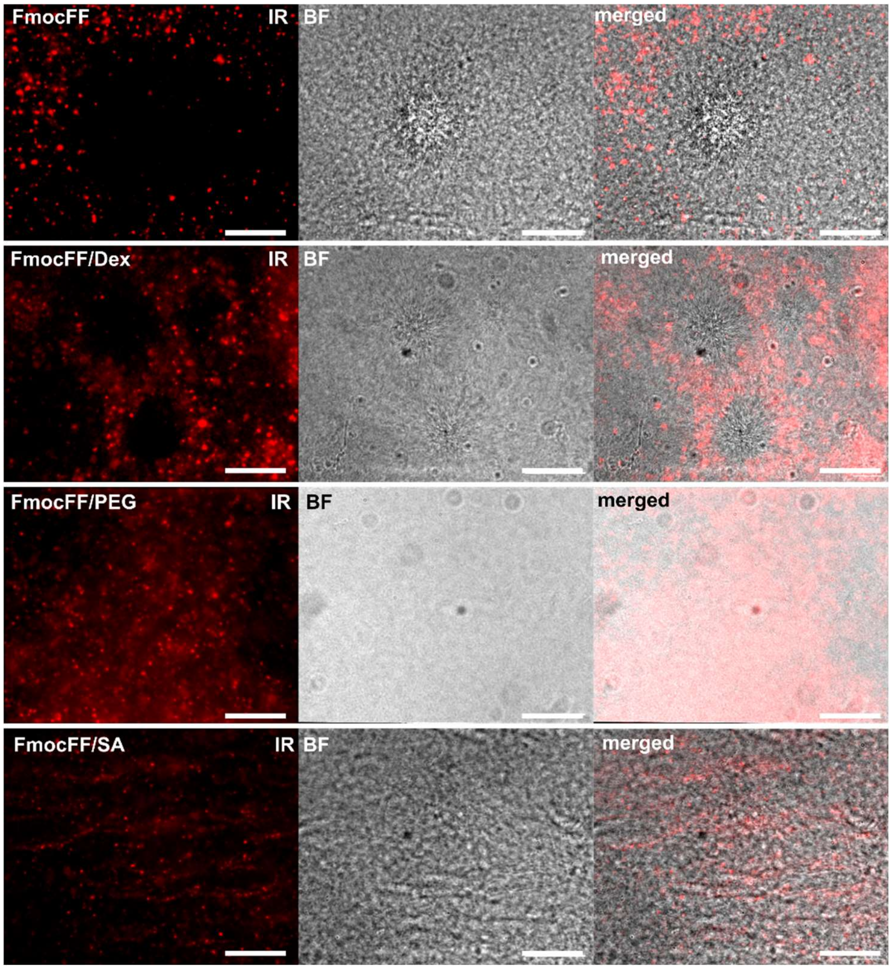

Figure S9: Fluorescence NIR-imaging of SWCNTs within FmocFF and FmocFF/polymer hydrogels after 24 h gelation time. Images showing examples for the NIR fluorescence channel (left), the respective bright-field image (middle), and the merged images (right) for the four hydrogels. Scale bar indicates 20 μm.

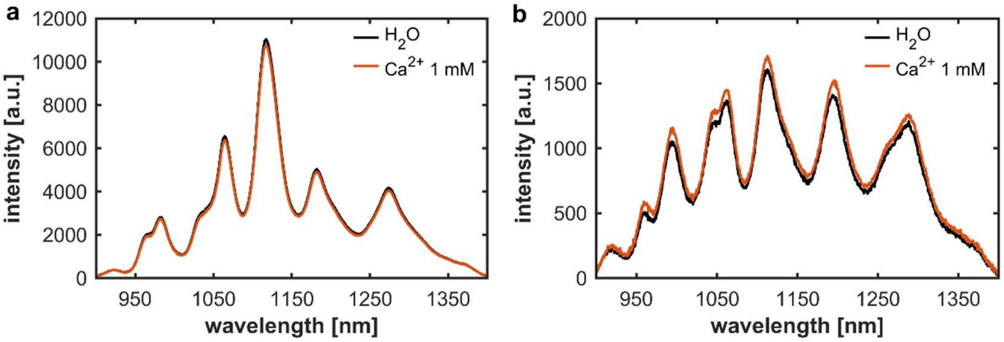

Figure S10: **Fluorescence spectrum of surfactant suspended SWCNT under addition of CA^{2+}.** a) sodium cholate suspended SWCNTs at a concentration of 0.5 mg L^{-1} after 2 h incubation with 1 mM Ca^{2+} (red) and the control spectrum after addition of water (black). b) sodium dodecylsulfate suspended SWCNTs at a concentration of 0.5 mg L^{-1} after 2 h incubation with 1 mM Ca^{2+} (red) and the control spectrum after addition of water (black) No quenching of the SWCNT-fluorescence was observed.

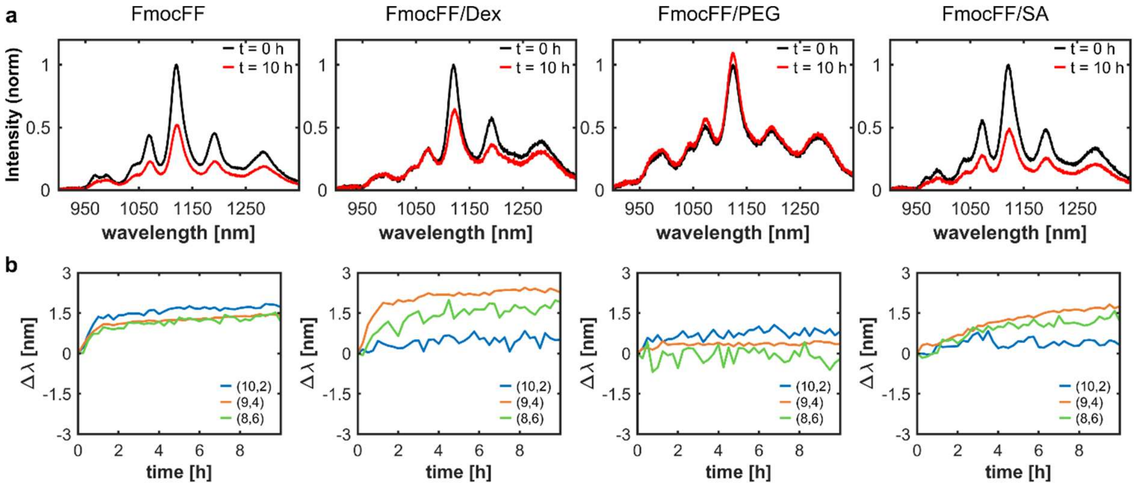

Figure S11: Normalized fluorescence spectra and wavelength shifts of SWCNTs integrated within FmocFF/polymer hydrogels upon addition of Ca^{2+}. a) Normalized fluorescence emission spectra of SWCNTs integrated within the hydrogels, before (black) and after (red) 10 h of incubation with Ca^{2+} 1 mM at an excitation wavelength of λ_{ex} = 730 nm. b) Time-dependent fluorescence wavelength shifts after addition of 1 mM Ca^{2+} of three different SWCNT chiralities: blue – (10,2); orange – (9,4); green – (8,6).

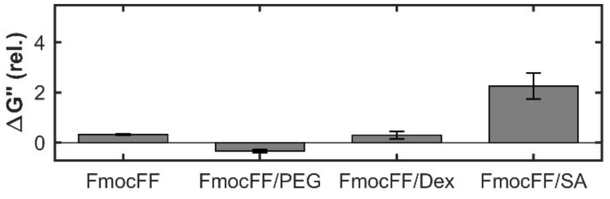

Figure S12: Changes in loss moduli after addition of 1 mM Ca^{2+} to the equilibrated hydrogels. Error bars show the mean and standard deviation (n = 3). The small decrease in G" for FmocFF/PEG is attributed to the hydration of the gels during the addition of Ca^{2+}-solution.

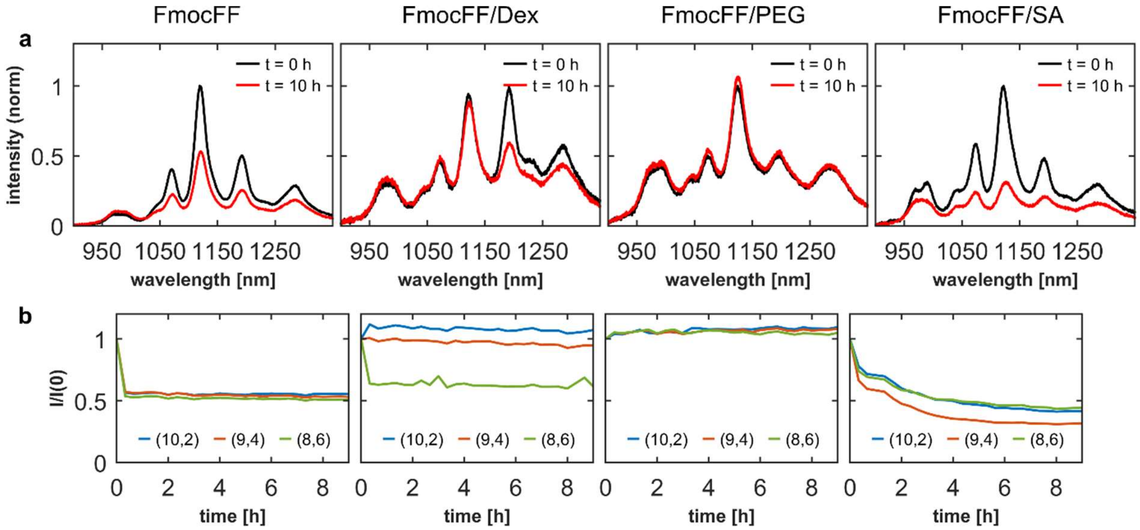

Figure S13: Normalized fluorescence spectra and wavelength shifts of SWCNTs integrated within one week old FmocFF/polymer hydrogels upon addition of Ca^{2+}. a) Normalized fluorescence emission spectra of SWCNTs integrated within the hydrogels, before (black) and after (red) 10 h of incubation with Ca^{2+} 1 mM at an excitation wavelength of λ_{ex} = 730 nm. b) Time-dependent fluorescence intensity changes after addition of 1 mM Ca^{2+} of three different SWCNT chiralities: blue – (10,2); orange – (9,4); green – (8,6). After one week of aging time, we observe small differences in the SWCNT spectra compared to the initial fluorescence of the SWCNT within the fresh gels. We attribute these changes to the aging of the hydrogels affecting the SWCNT fluorescence.

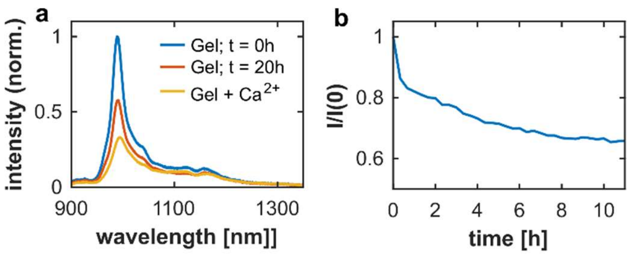

Figure S14: SWCNT sample with an enriched (6,5) chirality in FmocFF/SA hydrogels. a) Normalized SWCNT spectra measured at an excitation wavelength of λ_{ex} = 560 nm inside the hydrogels at t = 0 h (blue), after 20 h of gelation time (red) and after Ca^{2+} addition (yellow). b) Time dependent intensity decrease after the addition of Ca^{2+} to the FmocFF-hydrogels containing (6,5)-SWCNTs.

Supporting videos:

video1-FmocFF-1h.avi

video2-FmocFF-Dex-1h.avi

video3-FmocFF-PEG-1h.avi

video4-FmocFF-SA-1h.avi

video5-FmocFF-5h.avi

video6-FmocFF-Dex-5h.avi

video7-FmocFF-PEG-5h.avi

video8-FmocFF-SA-5h.avi

video9-FmocFF-24h.avi

video10-FmocFF-Dex-24h.avi

video11-FmocFF-PEG-24h.avi

video12-FmocFF-SA-24h.avi

References

(1) Tinevez, J. Y.; Perry, N.; Schindelin, J.; Hoopes, G. M.; Reynolds, G. D.; Laplantine, E.; Bednarek, S. Y.; Shorte, S. L.; Eliceiri, K. W. TrackMate: An Open and Extensible Platform for Single-Particle Tracking. *Methods* **2017**, *115*, 80–90.
